# Morphine Protects against Methylmercury Intoxication: A Role for Opioid Receptors in Oxidative Stress?

**DOI:** 10.1371/journal.pone.0110815

**Published:** 2014-10-17

**Authors:** Allan Costa-Malaquias, Mauro B. Almeida, José R. Souza Monteiro, Barbarella de Matos Macchi, José Luiz M. do Nascimento, María Elena Crespo-Lopez

**Affiliations:** 1 Laboratório de Farmacologia Molecular, Instituto de Ciências Biológicas, Universidade Federal do Pará (UFPA), Belém, Brasil; 2 Laboratório de Neuroquímica Molecular e Celular, Instituto de Ciências Biológicas, Universidade Federal do Pará (UFPA), Belém, Brasil; Indian Institute of Toxicology Reserach, India

## Abstract

Mercury is an extremely dangerous environmental contaminant responsible for episodes of human intoxication throughout the world. Methylmercury, the most toxic compound of this metal, mainly targets the central nervous system, accumulating preferentially in cells of glial origin and causing oxidative stress. Despite studies demonstrating the current exposure of human populations, the consequences of mercury intoxication and concomitant use of drugs targeting the central nervous system (especially drugs used in long-term treatments, such as analgesics) are completely unknown. Morphine is a major option for pain management; its global consumption more than quadrupled in the last decade. Controversially, morphine has been proposed to function in oxidative stress independent of the activation of the opioid receptors. In this work, a therapeutic concentration of morphine partially protected the cellular viability of cells from a C6 glioma cell line exposed to methylmercury. Morphine treatment also reduced lipid peroxidation and totally prevented increases in nitrite levels in those cells. A mechanistic study revealed no alteration in sulfhydryl groups or direct scavenging at this opioid concentration. Interestingly, the opioid antagonist naloxone completely eliminated the protective effect of morphine against methylmercury intoxication, pointing to opioid receptors as the major contributor to this action. Taken together, the experiments in the current study provide the first demonstration that a therapeutic concentration of morphine is able to reduce methylmercury-induced oxidative damage and cell death by activating the opioid receptors. Thus, these receptors may be a promising pharmacological target for modulating the deleterious effects of mercury intoxication. Although additional studies are necessary, our results support the clinical safety of using this opioid in methylmercury-intoxicated patients, suggesting that normal analgesic doses could confer an additional degree of protection against the cytotoxicity of this xenobiotic.

## Introduction

Mercury is a dangerous environmental contaminant. Methylmercury (MeHg), the most toxic compound of this metal, easily crosses biological membranes and tends to accumulate in tissues [1], [2]. This characteristic causes MeHg to bioaccumulate through the food chain, with the highest concentrations found in human populations, leading to the mercury intoxication events that have been observed throughout the world, for example in Japan, Seychelles, and the Amazon [3]–[5]. In the Amazon, environmental and human epidemiological studies demonstrated the chronic exposure of populations to this metal and revealed that the present levels of MeHg exceed the safety limits recommended by the World Health Organization [3], [6]. Consequently, environmental contamination with this heavy metal remains a serious challenge to Brazilian public health.

The central nervous system is the main target organ of this xenobiotic. The symptoms of acute human MeHg poisoning include ataxy, disartry, paresthesia, constraints of the visual field, mnemic deficits, and disturbances in the somatic sensory and hearing systems [7]. In addition, exposure to relatively low levels of MeHg can cause genotoxicity and delayed psychomotor development [8]–[11]. After reaching the central nervous system, MeHg elicits numerous cytotoxic events, particularly in glial cells that accumulate the highest concentrations of this metal [12], as these cells provide additional protection for neurons. A previous study revealed a key role for astrocytes in mediating MeHg neurotoxicity via accumulating MeHg and participating in the inhibition of neurotransmitters and the transport of metabolites, among other actions [13].

The molecular mechanisms elicited by MeHg exposure are not fully elucidated, but oxidative stress is closely related to toxicity and the neurodegeneration processes of mercury intoxication [7], [14]. Oxidative stress is an imbalance in redox homeostasis that results in the overproduction of free radicals (reacting molecules with an unpaired electron) such as nitric oxide, the major reactive nitrogen species. MeHg induces the activity of nitric oxide synthase, increasing the generation of nitric oxide [15] and provoking oxidative damage to biomolecules such as DNA, proteins, and lipids. Oxidative insults to lipids trigger the self-propagating process of lipid peroxidation, which especially affects membrane permeability (an initial event in mercury intoxication); enzymatic (superoxide dismutase and catalase, among others) and non-enzymatic (for example, glutathione) antioxidant systems prevent this damage. Glutathione is a major scavenger molecule, since it is the most common (up to mM concentrations in most cells) low molecular-weight compound containing sulfhydryl groups in mammalian cells [16]. These sulfhydryl groups indirectly indicate the redox state of the cell.

Oxidative stress also underlies other physiological responses, such as those elicited during drug treatments. However, the consequences of mercury intoxication and concomitant use of drugs targeting the central nervous system are completely unknown. This issue is especially important for drugs used in long-term treatments, such as analgesics. Morphine is one of the most potent analgesics, and possesses a recently demonstrated role in oxidative stress at therapeutic concentrations [17]. Morphine, an opioid, is commonly used to manage moderate to severe pain and is a major option in clinical practice [18]. The most recent report from the International Narcotics Control Board of the United Nations indicated a significant increase in the global consumption of morphine between 1992–2011, from 10 to 42 tons throughout the world [19].

The analgesic effect of morphine is attributed to activation of a family of metabotropic receptors (the µ-, δ-, and κ-type opioid receptors). Activation of the µ receptor, which is mainly responsible for morphine's effects, leads to the inhibition of calcium influx in pre-synaptic neurons and to an increase in potassium conductance in post-synaptic neurons, among other molecular effects. In addition to neurons, opioid receptors are localized in cells of glial origin, especially microglia and astrocytes, a feature that is preserved in gliomas like the C6 cell line [20].

A growing body of evidence suggests that morphine is active in oxidative stress, but despite morphine's extensive use, this role remains unclear and controversial [21]–[30]. Curiously, previous studies showing the antioxidant action of this opioid were carried out in isolated structures (mitochondria), in the presence of antagonists of opioid receptors, or in the presence of the synthetic enantiomer d-morphine (which does not bind the µ-opioid receptor), demonstrating that this antioxidant effect is independent of the opioid receptors [21], [23], [25]. Selective ligands for the µ-, δ-, and κ-type opioid receptors did not display any of the neuroprotective effects of morphine [21], [23]. Taken together, the results of these previous studies lead to the hypothesis that one or more molecular mechanisms may underlie the antioxidant activity of morphine: direct scavenger activity [21], [22], recovery of gluthathione levels [23], [27], and/or inhibition of NADPH oxidase [25]. Further, the effect of morphine in mercury intoxication remains totally unknown. Therefore, the aim of the current work was to analyze the effect of a therapeutic concentration of morphine on oxidative stress induced by MeHg exposure.

## Materials and Methods

### Cell culture

The rat glioma C6 cell line was obtained from the American Type Culture Collection (Manassas, VA, USA) and maintained at 37°C and 5% CO_2_ in Dulbecco's Modified Eagle Medium (CULTILAB, São Paulo, Brazil) supplemented with 10% (v/v) fetal bovine serum (CULTILAB), penicillin (50 U/mL), and streptomycin (50 µg/mL). Approximately 1.5×10^5^ cells were seeded and maintained at 37°C for 24 h before exposure to MeHg, morphine, and/or naloxone.

### Treatments

MeHg chloride (4 mM), morphine sulfate (10 mg/mL), and naloxone (4 mg/mL) were diluted in Dulbecco's Modified Eagle Medium. Cells were then incubated for 24 h with 0–11 µM, 0–10 µM, and 0–2.5 µM (final concentrations) of MeHg, morphine, and naloxone, respectively, in serum-free medium to avoid possible interference with serum proteins (MeHg shows high affinity for protein sulfhydryl groups).

### Analysis of cellular viability

Cellular viability was quantified via the 4,5-dimethylthiazol-3,5-diphenyltetrazolium (MTT) method [31]. This technique is based on the reduction of MTT by mitochondria, yielding a purple, insoluble salt. After treatments, cells were incubated for 2 h with MTT (0.5 mg/mL in phosphate-buffered saline) at room temperature. Three hundred microliters of dimethylsulfoxide were added, and absorbance was recorded at 570 nm. Cell viability was expressed as percentage of control groups.

### Assay of nitrite levels

Levels of nitrites, a stable product of nitric oxide degradation, were analyzed with the Griess assay [32]. After treatments, culture media were collected and Griess reagent (0.1% N-(1-naphthyl) ethylenediame and 1% sulfanilamide in 2.5% phosphoric acid) was added (1∶1), followed by incubation for 10 min at room temperature. Absorbance was measured at 540 nm and compared to measurements of standard concentrations of sodium nitrite.

### Quantitation of lipid peroxidation

Levels of lipid peroxidation were analyzed with the thiobarbituric acid assay [33]. Aldehydes formed by oxidative damage of membranes react with thiobarbituric acid and generate a pink compound. Briefly, treated cells were homogenized in a solution containing 1% thiobarbituric acid and 25% trichloroacetic (w/v) in ultra-pure water. Homogenates were heated at 100°C for 45 min and centrifuged for 5 min at 150×*g*. Absorbance of the supernatants was recorded at 535 nm and compared to measurements of standard concentrations of malondialdehyde.

### Analysis of sulfhydryl groups

The levels of compounds containing sulfhydryl groups were assayed by the method described by Elman (1959) using the selective reaction of these groups with the acid 5,5′-dithium-bis(2-nitrobenzoic acid) (Elman's reagent) [34]. Treated cells were homogenized in ice-cold phosphate-buffered saline with 1 mM ethylenediaminetetraacetic acid and 0.1% sodium dodecyl sulfate and centrifuged for 5 min at 1500 rpm. Supernatants were treated for 5 min with developer solution (5 mM 5,5′-dithium-bis(2-nitrobenzoic acid)) and absorbance was evaluated at 412 nm. Absorbance values were compared to measurements of standard solutions of glutathione.

### Total protein content

Total protein content was determined in all samples as described elsewhere [35]. After treatments, cells were homogenized in ultra-pure water and incubated with Bradford reagent (5% ethanol, 8.5% phosphoric acid, 0.25% Coomassie Brilliant Blue G-250) for 2 min at room temperature. The absorbance of each sample was measured at 595 nm and compared to measurements of standard solutions of bovine serum albumin. Values of lipid peroxidation, nitrites, and sulfhydryl groups were, then, corrected for protein concentration of each sample and, finally, they were expressed as percentages of control groups.

### Assay of direct scavenging of free radicals

To evaluate the possibility of direct scavenging of free radicals by morphine, the protocol described by Gülçin et al. [22] was carried out. Briefly, morphine (1–10 µM) was added to a solution of 40 µg/mL 1,1diphenyl-2-picrylhydrazyl (DPPH·) in ethanol. After 30 min, absorbance was measured at 517 nm and the percentage of scavenged DPPH molecules was calculated according to the formula:
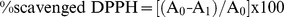
where A_0_ is the absorbance of the control group and A_1_ is the absorbance in the presence of morphine.

### Statistical analysis

Statistical analysis was performed with BIOESTAT 5.3 (free available at: http://www.mamiraua.org.br/pt-br/downloads/programas/). Analysis of variance was carried out, followed by a *post hoc* Tukey's test when appropriate. Differences with p<0.05 were considered significant.

## Results

### Viability of C6 cells decreased in a concentration-dependent manner after MeHg exposure

**Figure 1:**
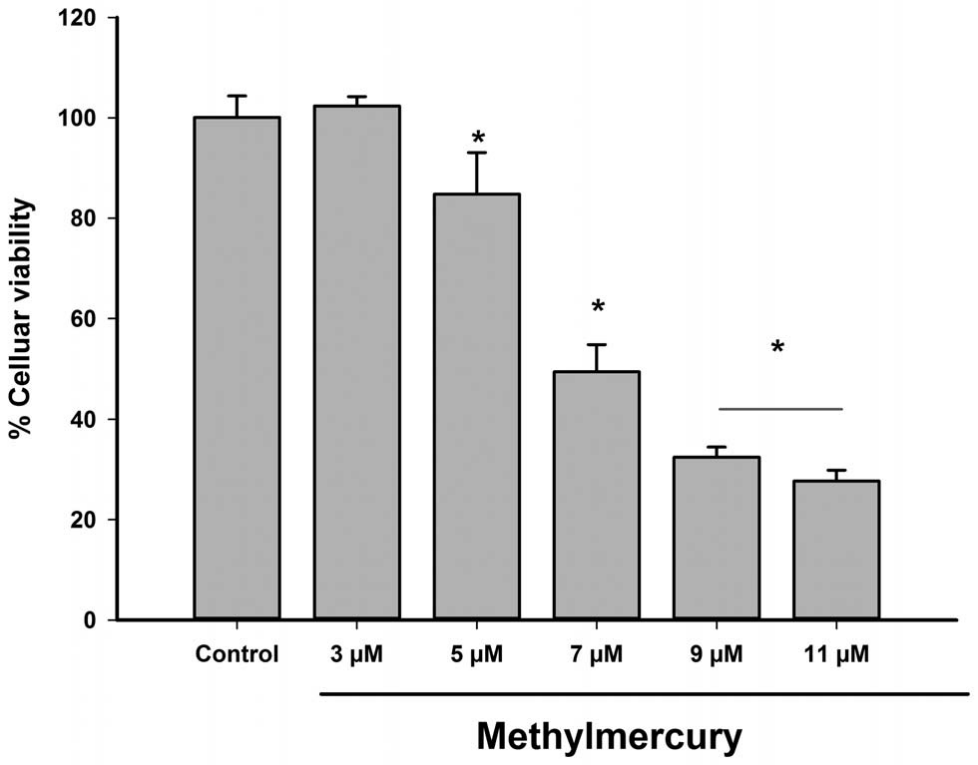
Viability of C6 cells exposed to increasing concentrations of methylmercury (MeHg) for 24 h. Data are expressed as mean ± standard deviation (n = 4). *P<0.01 versus all groups.

After exposure to 0–11 µM MeHg for 24 h, C6 glioma cells displayed significantly decreased viability with MeHg concentrations of 5 µM or above, reaching a minimum of 28% initial viability with 11 µM (Figure 1). We fitted a concentration-response curve to a sigmoid curve to calculate the median lethal concentration for C6 cells (in this case, 6.96 µM). For subsequent experiments, 6 µM of MeHg was used.

### Morphine partially prevented the decrease in cell viability induced by MeHg exposure

**Figure 2:**
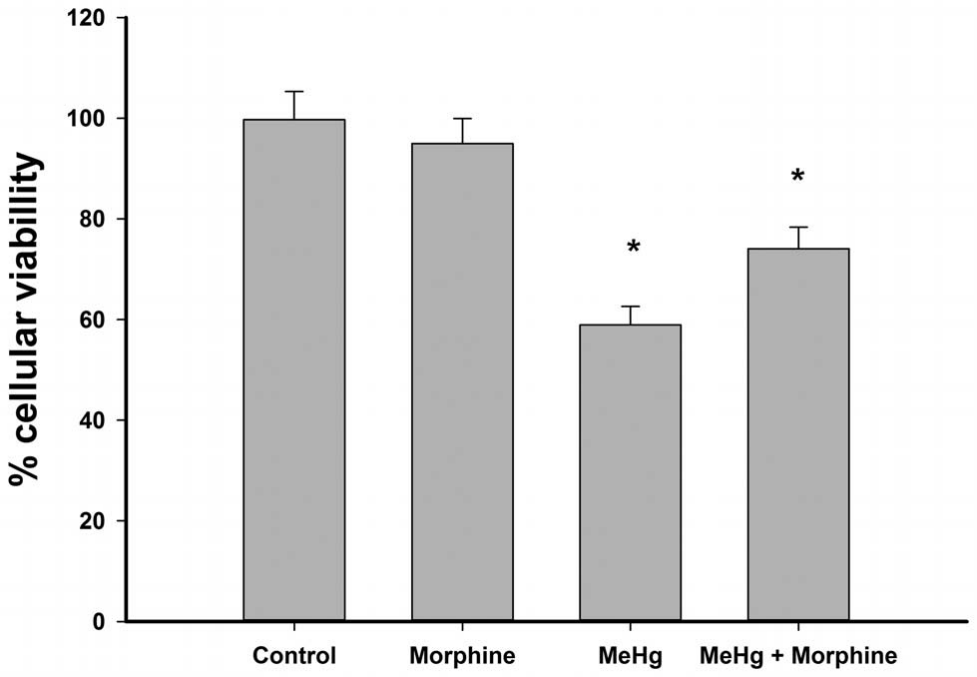
Viability of C6 cells incubated with 1 µM morphine and/or 6 µM methylmercury (MeHg) for 24 h. Data are reported as mean ± standard deviation (n = 3). *P<0.05 versus all groups.

Intoxication with 6 µM MeHg for 24 h significantly decreased C6 cellular viability by ∼40% (Figure 2); this deleterious effect was partially avoided by co-treatment with morphine (Figure 2). Exposure to morphine alone did not affect cellular viability (Figure 2).

### Morphine reduced nitrite levels in C6 glioma cells exposed to MeHg

**Figure 3:**
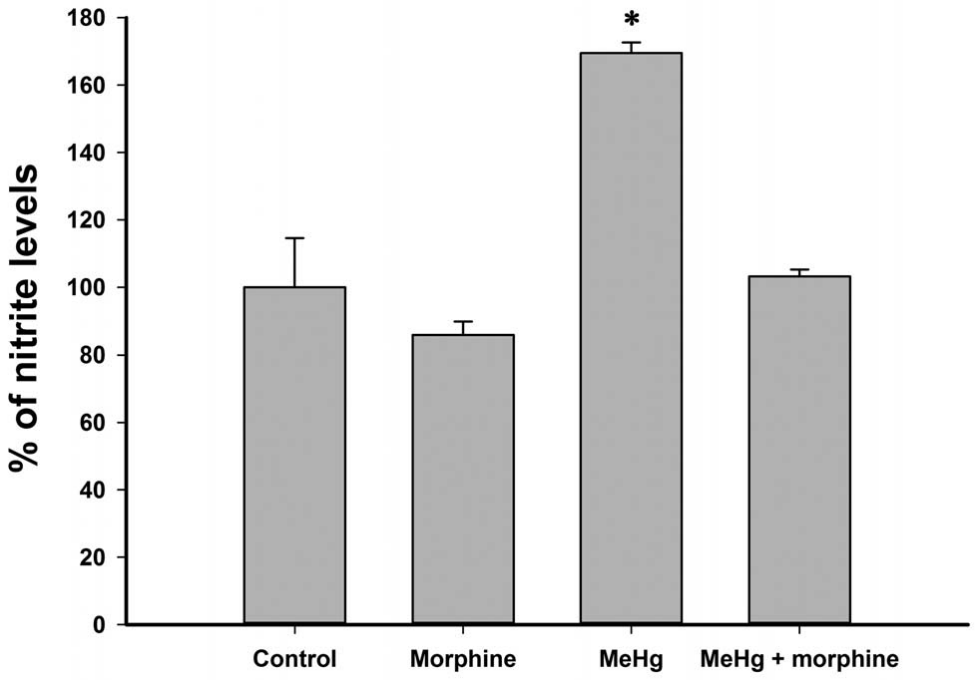
Nitrites in C6 cells exposed to 1 µM morphine and/or 6 µM methylmercury (MeHg) for 24 h. Data are shown as mean ± standard deviation (n = 3). *P<0.01 versus all groups.

To evaluate the ability of morphine to reduce nitric oxide overproduction, nitrite levels were quantified as an indirect parameter. MeHg exposure increased nitrite levels when compared to those of the control group (Figure 3). This increase was totally prevented by co-treatment with morphine, but morphine alone did not affect nitrite concentrations (Figure 3).

### Morphine reduced lipid peroxidation in C6 glioma cells

**Figure 4:**
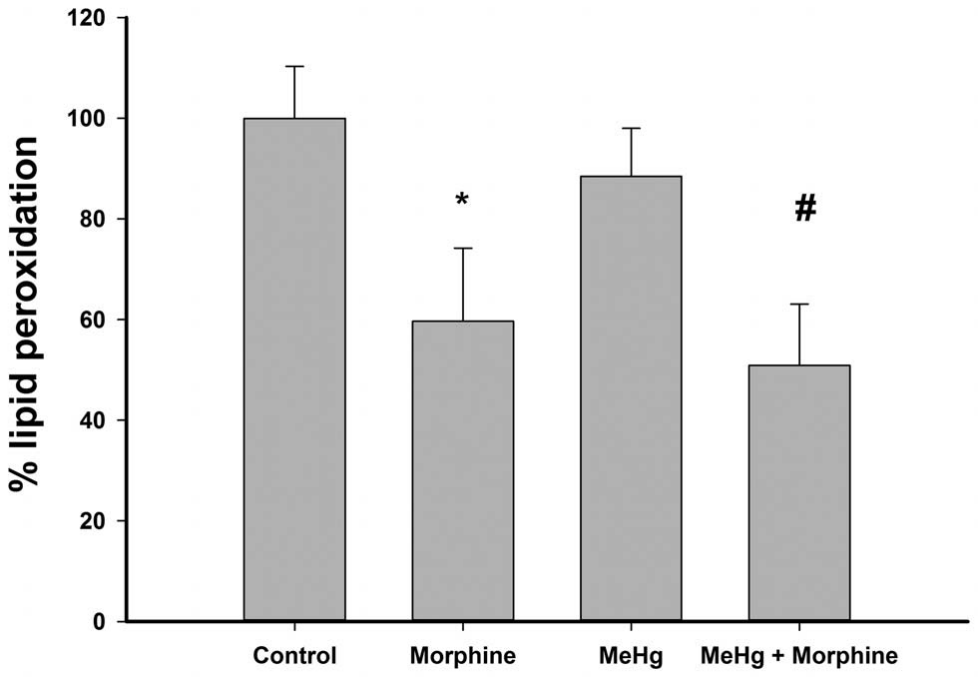
Lipid peroxidation in C6 cells incubated with 1 µM morphine and/or 6 µM methylmercury (MeHg) for 24 h. Data are reported as mean ± standard deviation (n = 3). *P<0.05 versus control group; #P<0.05 versus control and MeHg groups.

No significant differences in the levels of lipid peroxidation were detected between control and MeHg-exposed groups (Figure 4). However, all groups exposed to 1 µM morphine exhibited decreased levels of lipid peroxidation compared to those in the control and MeHg groups (Figure 4). When cells were incubated with medium supplemented with fetal bovine serum, lipid peroxidation was only 46.1±15.9% of the control group in Figure 4 (serum-free), indicating that absence of serum significantly increased lipid peroxidation (Student t test, P<0.05).

### Morphine exhibited a concentration-dependent scavenger effect

**Figure 5:**
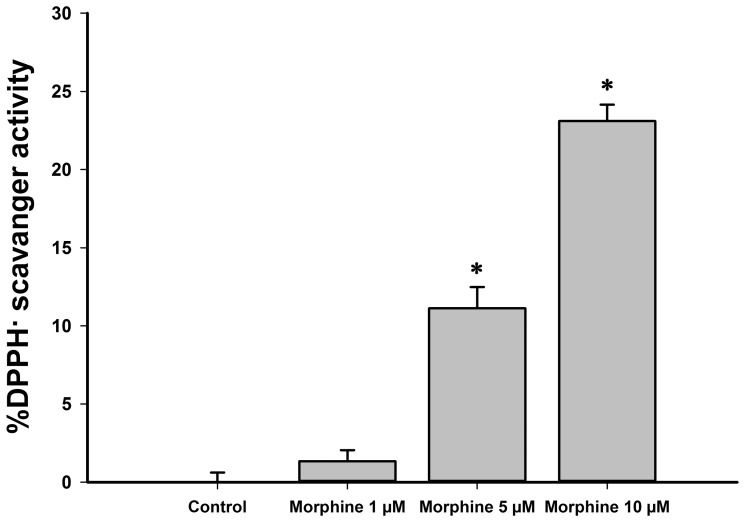
Direct scavenging activity of increasing concentrations of morphine against free radicals. Morphine was incubated with a 1,1diphenyl-2-picrylhydrazyl solution; results are shown as mean ± standard deviation (n = 9). *P<0.01 versus control group. ^#^P<0.01 versus all groups.

When a direct challenge with DPPH· was carried out, morphine neutralized this free radical in a concentration-dependent manner (Figure 5). However, this direct scavenger effect was only detected with ≥5 µM morphine.

### Neither morphine nor MeHg altered the levels of sulfhydryl groups in C6 cells

**Figure 6:**
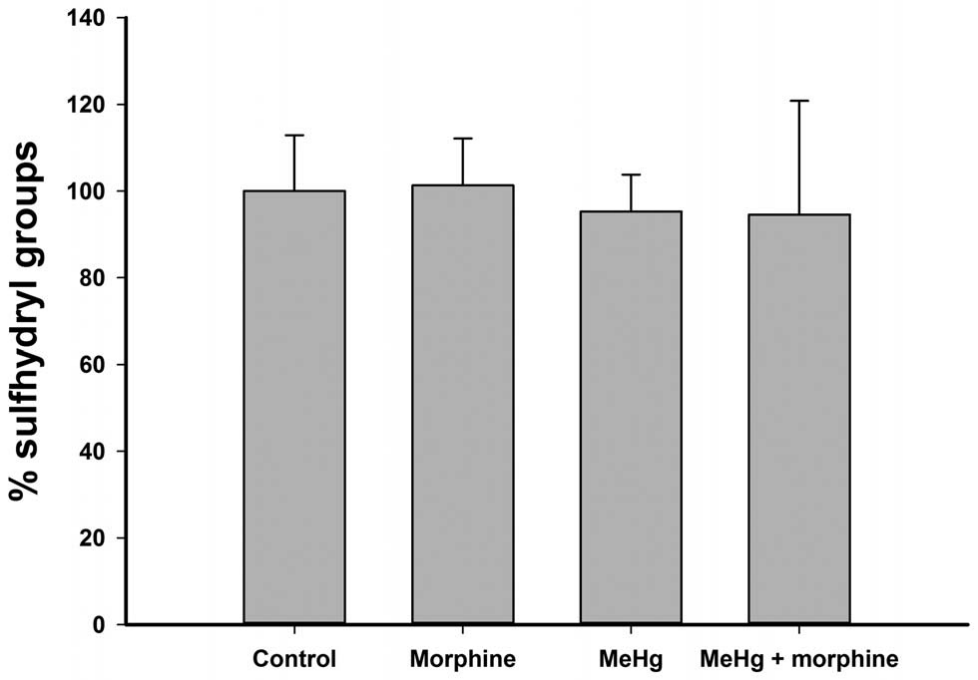
Sulfhydryl-group content in C6 cells exposed to 1 µM morphine and/or 6 µM methylmercury (MeHg) for 24 h. Data are expressed as mean ± standard deviation (n = 3). No significant differences were detected between groups.

The levels of sulfhydryl groups were similar for all treatments (Figure 6), and no significant changes were observed after 24 h of incubation.

### Treatment with increased concentrations of naloxone did not affect cellular viability in C6 cells

Due to the absence of previous investigations of the effect of naloxone, an opioid antagonist, we analyzed cellular viability after exposure to 0–2.5 µM naloxone to evaluate the interference of this drug. No significant difference was detected between groups (Figure S1). For subsequent experiments, the highest concentration of naloxone (2.5 µM) was chosen to assure that opioid receptors were totally blocked.

### Naloxone completely prevented the protective effect of morphine against MeHg intoxication

**Figure 7:**
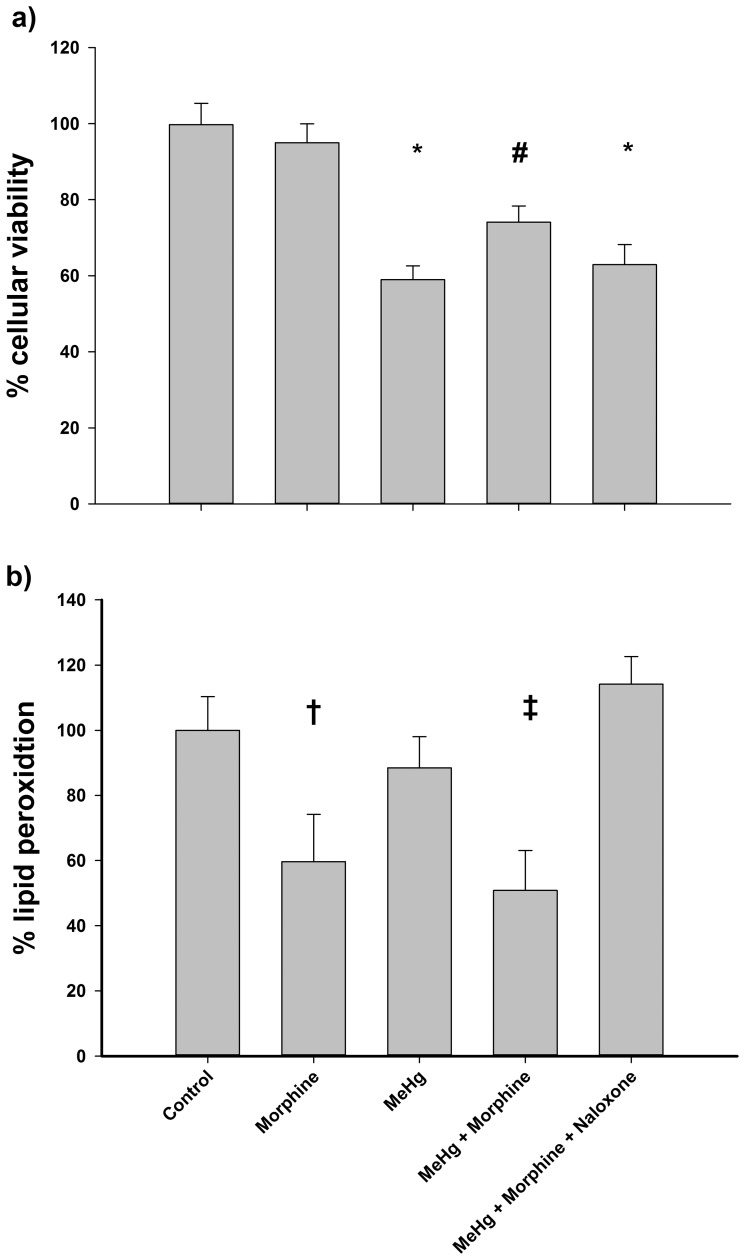
Cellular viability (a) and lipid peroxidation (b) after exposure to morphine, methylmercury (MeHg) and/or naloxone. C6 cells were incubated for 24 h with 1 µM morphine, 6 µM MeHg, and/or 2.5 µM naloxone (+ and − symbols indicate the presence or absence of each drug, respectively). Data are expressed as mean ± standard deviation. *P<0.05 versus control and morphine group. ^#^P<0.05 versus all groups. ^†^P<0.05 versus control and the group incubated with naloxone. ^‡^P<0.05 versus all groups except that incubated only with morphine.

To determine whether the protective action of morphine against MeHg was dependent on the activation of opioid receptors, co-treatment with naloxone was carried out. Under these conditions, the protective effect of morphine on cellular viability was completely abolished by naloxone (Figure 7a). Additionally, cells that underwent naloxone did not display the decreases in lipid peroxidation caused by morphine exposure (Figure 7b).

## Discussion

This work provides the first demonstration that a therapeutic concentration of morphine partially avoids the loss of cellular viability (Figure 2) and completely prevents the increase of nitrite levels (Figure 3) observed after exposure of glioma cells to MeHg. This study was carried out in C6 glioma cells due to the importance of glial cells in MeHg intoxication. The initial deleterious events observed in mercury poisoning are related to imbalances in glial cells; oxidative stress plays a crucial role in cell damage and death [13], [14]. Furthermore, this glial cell line has already been shown to be a good model for studying oxidative stress related to MeHg exposure [36]–[38].

In this work, a median lethal concentration of 6.96 µM was detected for MeHg intoxication (Figure 1). This value is between those previously described for glial cell lines and for primary cultures of astrocytes exposed to MeHg [9]–[39], being closer to the latter value. On the other hand, the morphine concentration used in our work was not cytotoxic to the C6 cell line (Figure 2), in agreement with previous reports showing that even higher concentrations of morphine do not significantly reduce cellular survival in C6 cells [17], [23].

Previous studies typically employed morphine doses that exceed the range of plasmatic concentrations (16–364 ng/mL) in patients undergoing chronic treatment for pain management in clinical practice [40]. The concentration of morphine used in the current work (1 µM, equivalent to 285.34 ng/mL) is just below the upper limit of the plasmatic concentrations in patients (high liposolubility of morphine allows transit through the blood brain barrier, making the morphine levels in nervous tissue very similar to those in blood). Employing concentrations similar to those used in clinical practice contributes to stronger comparisons between experimental models and the clinical setting. This therapeutic concentration of the opioid previously displayed a protective effect against oxidative damage caused by hydrogen peroxide [17]. However, the effects of morphine in a pathological situation such as mercury intoxication, in which oxidative stress is a major molecular mechanism, remain unknown.

Here, the use of a high concentration of MeHg decreased cellular viability to ∼60%, but the therapeutic concentration of morphine reduced this deleterious consequence by ∼15% (Figure 2). These results demonstrated for the first time that the protective effect of this opioid is potent enough to significantly affect cellular viability, including under the extreme conditions investigated here.

MeHg intoxication was associated with a pronounced increase in nitrite levels (Figure 3). This observation was consistent with previous demonstrations that MeHg exposure enhances nitric oxide production via the activation of nitric oxide synthase [14], [15]. Nitrite levels have been used as an indirect marker of nitric oxide production, which correlates with increased nitric oxide synthase activity under conditions of MeHg intoxication [41]. Interestingly, morphine totally prevented the increase in nitrite levels caused by MeHg (Figure 3).

Nitric oxide is a major free radical responsible for the oxidative damage resulting from MeHg exposure, leading to mitochondrial dysfunction and/or lipid peroxidation, among other effects [7], [42]. Thus, lipid peroxidation is considered to be an oxidative hallmark in MeHg-induced neurotoxicity [7], [42]. Curiously, we did not detect higher levels of lipid peroxidation following the exposure of C6 cells to MeHg (Figure 4). Our hypothesis is that in the control group, these levels may reach an upper limit due to the incubation for 24 h in serum-free medium; in the presence of serum, lipid peroxidation levels remained 54% lower than those of the control serum-free group (data not shown). This phenomenon was previously observed during oxidative stress with organic peroxides [43]. Cultures of C6 cells exposed to organic peroxides showed a concentration-dependent increase in lipid peroxidation levels up to a limit, after which increases in the concentration of organic peroxide did not alter lipid peroxidation levels, even when viability was reduced [43]. Nevertheless, all groups incubated with morphine exhibited significantly lower levels of lipid peroxidation than those measured in the control and MeHg groups (Figure 4). Taken together, these results point to a potent antioxidant action of morphine. Although the role of this opioid in oxidative stress was controversial in previous works, here we demonstrate for the first time that morphine exerts a neuroprotective effect during MeHg intoxication.

What molecular mechanisms underlie this antioxidant action? To date, four possibilities were hypothesized for the antioxidant effect of morphine: 1) direct scavenger effect, 2) changes in levels of glutathione, 3) changes in activity of NADPH oxidase and 4) activation of opioid receptors (see [17]). Interestingly, our analysis of possible molecular mechanisms revealed no significant scavenger effect with 1 µM morphine (Figure 5). Although direct scavenging was previously demonstrated for higher morphine concentrations [22], we recently demonstrated that 1 µM morphine did not scavenge molecules of hydrogen peroxide in a direct challenge [17]. Our suspicion of the absence of scavenging at this therapeutic morphine concentration was confirmed in the present work; morphine eliminated DPPH radicals only at concentrations of 5 µM or more (Figure 5).

No change in the levels of sulfhydryl groups was detected for any group (Figures 6). The high affinity of MeHg for sulfhydryl groups provokes the binding of the metal to reduced glutathione, the major intracellular compound containing these groups. The resulting complex is easily excretable and usually results in the intracellular depletion of glutathione (and consequently, the depletion of sulfhydryl groups). This depletion was previously observed following MeHg exposure [44],[45], but we did not detect it here. A possible explanation for this discrepancy could be a recovery of the levels of sulfhydryl groups after 24 h of incubation with the metal, consistent with previous observations [46], but additional studies must be carried out to confirm this hypothesis.

Alterations in glutathione levels following morphine treatment are frequently observed even at higher concentrations of the opioid [24], [26], [29], [30]. Previous experiments demonstrated a protective role of the opioid via increasing concentrations of sulfhydryl groups in both astrocytes and C6 cells exposed to glutamate [23]. However, our observations (no change in sulfhydryl groups with morphine treatment; Figure 6) are consistent with a previous study [17] demonstrating that this therapeutic concentration of morphine may not affect sulfhydryl groups. Taken together, these results suggest that glutathione levels are probably not a critical factor in morphine protection against MeHg exposure.

Based on similar results obtained with a classical model of oxidative stress using hydrogen peroxide exposure [17], we recently suggested that a possible receptor-mediated action of morphine in oxidative stress should be not discarded. Thus, to assess the role of opioid receptors in morphine's antioxidant protection, we investigated the effects of naloxone, an opioid antagonist. Interestingly, the protective action of morphine was completely abolished in the presence of the antagonist (Figure 7). Both cellular viability (Figure 7a) and the levels of lipid peroxidation (Figure 7b) were similar to those measured in cells exposed to MeHg. These results point to an essential role for opioid receptors (at least for the µ- and κ-type receptors that are present in C6 cells [20]) in the mediation of this effect in cells of glial origin.

In conclusion, this study demonstrated, for the first time, that a therapeutic concentration of morphine reduces the oxidative damage and cell death caused by MeHg intoxication through the activation of opioid receptors. Thus, these receptors may be a promising pharmacological target for decreasing oxidative damage and cell death after mercury intoxication. Although more studies are necessary, these results support the clinical safety of using morphine in MeHg-intoxicated people, suggesting that normal analgesic doses could confer an additional degree of protection against the cytotoxicity of this xenobiotic.

## Supporting Information

Figure S1 **Cellular viability of C6 cell line exposed to increased concentrations of morphine for 24 h.** No significant difference was detected between groups.
(TIF)
