# Supplementary figures and images for: Morphine Protects against Methylmercury Intoxication: A Role for Opioid Receptors in Oxidative Stress?
Source: PLoS One. 2014 Oct 17;9(10):e110815. doi: 10.1371/journal.pone.0110815 (PMC4201572; doi:10.1371/journal.pone.0110815)

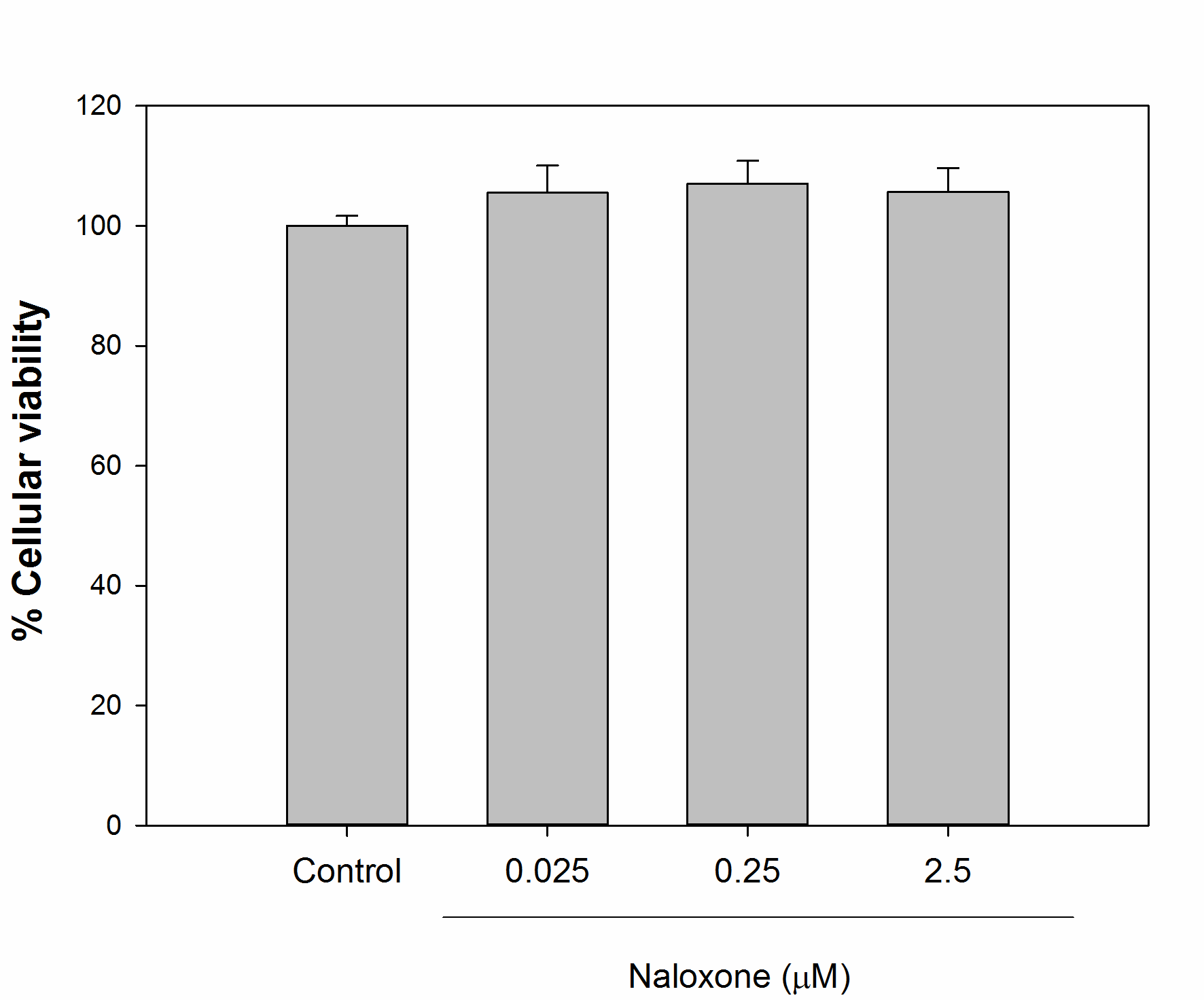

Supplement: Figure S1 — Cellular viability of C6 cell line exposed to increased concentrations of morphine for 24 h. No significant difference was detected between groups. (TIF) [file pone.0110815.s001.tif]
